# An epidemiological model for mosquito host selection and temperature-dependent transmission of West Nile virus

**DOI:** 10.1038/s41598-022-24527-5

**Published:** 2022-11-19

**Authors:** Augusto Fasano, Nicola Riccetti, Anastasia Angelou, Jaime Gomez-Ramirez, Federico Ferraccioli, Ioannis Kioutsioukis, Nikolaos I. Stilianakis

**Affiliations:** 1 Joint Research Centre (JRC), European Commission, Via E. Fermi 2749, 21027 Ispra, VA Italy; 2 Department of Physics, University of Patras, 26504 Rio, Greece; 3 Department of Biometry and Epidemiology, University of Erlangen-Nuremberg, Waldstraße 6, 91054 Erlangen, Germany

**Keywords:** Ecological epidemiology, Ecological modelling, Infectious diseases, Computational science

## Abstract

We extend a previously developed epidemiological model for West Nile virus (WNV) infection in humans in Greece, employing laboratory-confirmed WNV cases and mosquito-specific characteristics of transmission, such as host selection and temperature-dependent transmission of the virus. Host selection was defined by bird host selection and human host selection, the latter accounting only for the fraction of humans that develop symptoms after the virus is acquired. To model the role of temperature on virus transmission, we considered five temperature intervals (≤ 19.25 °C; > 19.25 and < 21.75 °C; ≥ 21.75 and < 24.25 °C; ≥ 24.25 and < 26.75 °C; and > 26.75 °C). The capacity of the new model to fit human cases and the week of first case occurrence was compared with the original model and showed improved performance. The model was also used to infer further quantities of interest, such as the force of infection for different temperatures as well as mosquito and bird abundances. Our results indicate that the inclusion of mosquito-specific characteristics in epidemiological models of mosquito-borne diseases leads to improved modelling capacity.

## Introduction

West Nile virus (WNV) is a *Flavivirus* maintained in nature in a complex enzootic cycle between mosquitoes (mainly within the genus *Culex*) and birds (mainly within the order Passeriformes)^1–5^. Mammals, including humans, can occasionally become infected with WNV. However, due to their inability to develop sufficient viremia to reinfect the mosquitoes (predominantly *Culex*), mammals are considered dead-end hosts. Similarly, low viremias are also present in several bird species (e.g., domestic chickens), rendering not all avian populations a possible source of infection for mosquitoes^6–9^.

WNV infections in humans are largely asymptomatic (about 80%)^10^. The apparent infection can present either as an influenza-like syndrome (West Nile fever [WNF]), which often includes a cutaneous rash, or, more rarely, as a neuroinvasive syndrome with meningitis/encephalitis, flaccid paralysis and a 10% case fatality rate (West Nile neuroinvasive disease [WNND])^1^. Approximately 1% develop severe symptoms (WNND)^10^.

Human WNV cases often occur with a specific seasonality, starting in summer and peaking in late summer/early autumn^11^. Several authors have speculated that this specific seasonality might be the result of a shift in mosquito feeding patterns^12–14^. This shift might be due to mosquito-specific aspects, such as higher vector density in late summer than in early spring and/or different time points in the peak of the different *Culex* populations, each with a different host spectrum^14^. Host-specific aspects might also play a role. The different availability of hosts, both in the sense of higher/easier availability (e.g., nestling/nesting parents) or lower availability (e.g., emigration of preferred host species)^12,15^, and the development of stronger defensive behaviours by potential hosts might also be a cause for the shift in feeding patterns^16^. These host-specific aspects become relevant when considering that all WNV-transmitting *Culex* mosquitoes have been observed to have opportunistic feeding behaviours and thus might switch to more mammal-derived blood meals in case of need.

In addition, mosquito populations and their ability to spread WNV show a certain seasonal pattern. Warmer temperature and the lack of precipitation and/or wind represent a more favourable environment for WNV-transmitting mosquitoes (e.g., higher rate of oviposition, lower mortality rates)^17,18^ and therefore greater trapping success^19^. In addition, although largely influenced by midgut infection barriers and virus replication^20^, infection and transmission rates by *Culex* mosquitoes may also be influenced by temperature^21^. These effects also may lead to the frequently documented association between warmer temperatures and precipitation and the incidence of WNV cases among humans^22,23^.

Epidemiological models have been used to estimate the best moment for intervention, to predict human WNV cases and to understand the minimum set of information needed to predict cases with accuracy. WNV models have become more complex to include the aforementioned effects of temperature and precipitation as well as landscape features available from satellite Earth observation data^19,24^ on the population dynamics of mosquitoes and therefore on the amplification and transmission of the virus^25,26^. In this context, and following previous modelling work by Hartley et al.^27^, epidemiological models can be used to gain further understanding of the complex cycle of transmission of WNV. For these reasons, we included host selection and temperature-dependent transmission in our previously developed weather-dependent spatial epideMIological ModEl for WNV tranSmISsion (MIMESIS)^26^.

Our aim was to extend the MIMESIS model for WNV infection in humans to further describe mosquito-specific characteristics of WNV transmission, including biological and epidemiological aspects.

## Results

**Figure 1:**
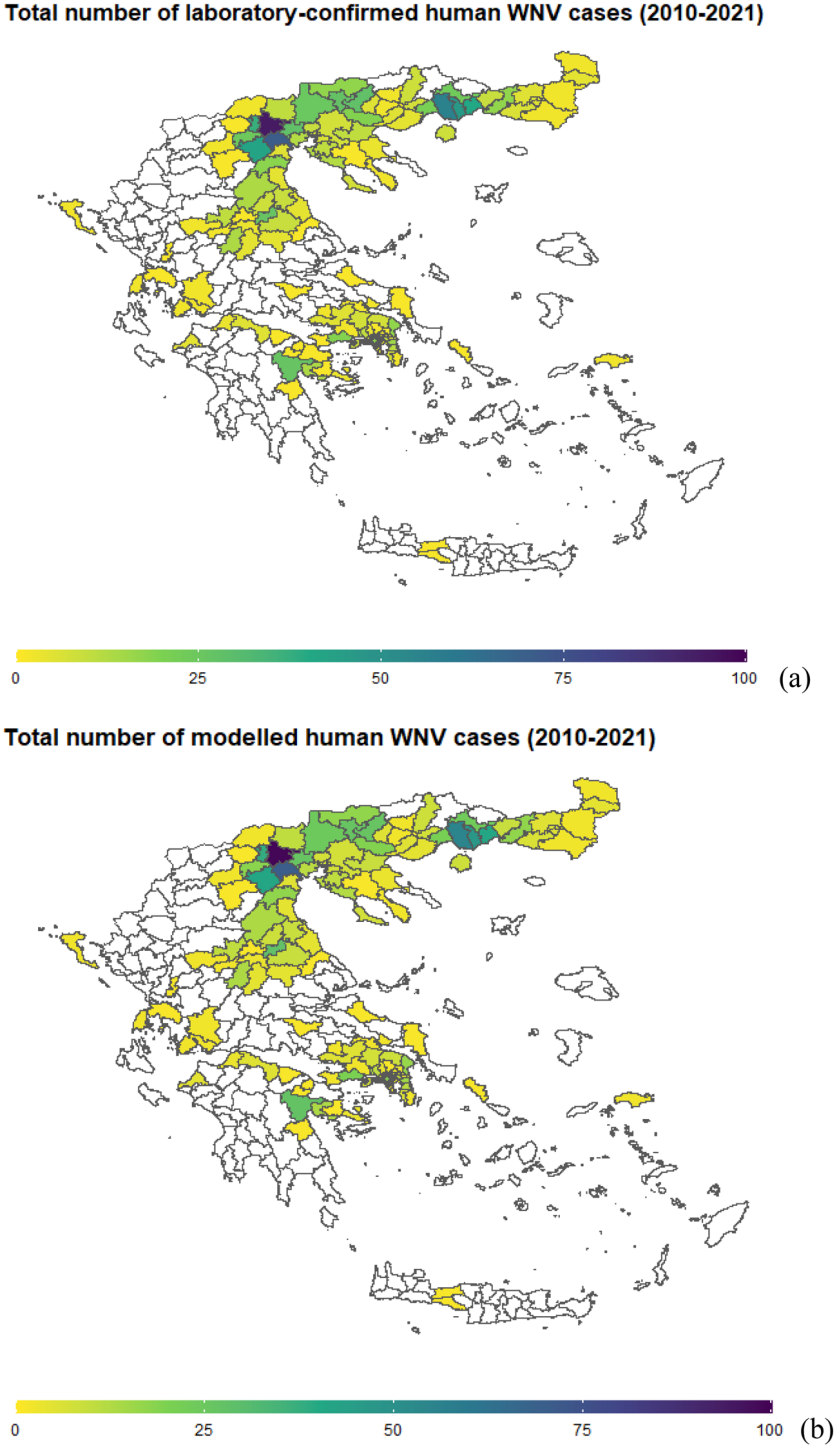
(**a**) Map of Greece with total numbers of laboratory-confirmed human WNV cases throughout the 12-year period 2010–2021, with breakdowns by municipality. White denotes municipalities where no human cases were observed. (**b**) Map of Greece with total numbers of modelled human WNV cases throughout the 12-year period 2010–2021, with breakdowns by municipality. White denotes municipalities where no human cases were modelled.

Among the 325 municipalities in Greece during the period 2010–2021, WNV events, defined as the occurrence of at least one laboratory-confirmed human WNV case during a specific year, were reported in 154 (47%) municipalities, while the remaining 171 did not report any WNV case. WNV events were reported for a period ranging from one to eight years: 54 (35%) municipalities reported laboratory-confirmed WNV cases in only one year, 38 (25%) in two years, 30 (19%) in three years, 12 (8%) in four years, 10 (6%) in five years, 6 (4%) in six years, 1 (1%) in seven years, and 3 (2%) in eight years. This means that in 60% of the positive areas (82 municipalities out of 154), WNV appeared at most for two years, in 27% (42 out of 154) between three and four years, and in the remaining 13% (20 out of 154) for five years or more. Considering the total number of reported laboratory-confirmed human WNV cases across the twelve years (Fig. 1), in approximately 50% of the positive municipalities (78 out of 154), at most 4 cases were reported: 1, 2, 3, and 4 WNV cases were reported in 24, 32, 11, and 11 municipalities, respectively. Overall, 39 municipalities recorded a number of WNV cases ranging from 5 to 10 (third quartile), 34 a number ranging from 11 to 46, while the remaining 3 municipalities recorded a number of WNV cases equal to 56, 71 and 94.

### Model evaluation and comparison with MIMESIS

We investigated the ability of the MIMESIS-2 model to correctly identify the occurrence of WNV events, both in space and time, and its capacity to quantify the annual number of human WNV cases and the timing of the first WNV event in the year. The performance of many quantities of interest, such as the severity and timing of occurrence of human WNV cases, was also compared output from the original MIMESIS model^26^.

### Occurrence of WNV events

Starting with the spatial analysis, we considered the fit of the model to replicate the observed 385 WNV events out of 3,900 (325*12) possible events across municipalities. MIMESIS-2 was able to correctly identify 356 of them, generated only one false alarm, and correctly modelled 3,514 true negatives.

The performance of MIMESIS-2 was then evaluated according to four indices: the probability of detection (POD), false alarm rate (FAR), miss rate (MIS), and critical success index (CSI), described in the Methods section. For the POD, MIS and CSI, we considered the 154 municipalities with at least one reported and laboratory-confirmed human WNV case over the 12-year period, while for the FAR, we considered the 153 municipalities where at least one human WNV case was modelled over the same period. We split the (0.0–1.0) index interval into five equally sized bins to derive for each index, the fraction of municipalities falling into each bin. Both the POD and CSI were above 0.8 for 139 municipalities out of 154, while the MIS was below 0.2 for 142 municipalities (out of 154) and the FAR was always below 0.2, with one false alarm produced in a municipality where WNV events were observed in eight out of twelve years (Table 1).

**Table 1:** Capacity of the MIMESIS-2 model to correctly model laboratory-confirmed human WNV cases.

%	POD^§^	FAR^§§^	MIS^§§§^	CSI^§§§§^
0–20	1	153	142	1
21–40	0	0	8	0
41–60	3	0	3	3
61–80	11	0	0	11
81–100	139	0	1	139
SUM	154	153	154	154

Included are the absolute number of municipalities from each of the examined indices (POD, FAR, MIS, CSI) and each accuracy zone.

^§^$$\mathrm{POD}=\mathrm{Hits}/(\mathrm{Hits}+\mathrm{Misses})$$.

^§§^$$\mathrm{FAR}=(\mathrm{False Alarms})/(\mathrm{Hits}+\mathrm{False Alarms})$$.

^§§§^$$\mathrm{MIS}=\mathrm{ Misses}/(\mathrm{Hits}+\mathrm{Misses})$$.

^§§§§^$$\mathrm{CSI}=\mathrm{Hits}/(\mathrm{Hits}+\mathrm{False Alarms}+\mathrm{Misses})$$.

with Hits (municipalities with cases both observed and simulated for a specific year),

False alarms (municipalities with cases simulated but not observed for a specific year), and.

Misses (municipalities with cases observed but not simulated for a specific year).

We also analysed how the model performed in different years by studying the multiannual evolution of the indices. Both the aggregated POD and CSI were equal to 0.92, with annual variations ranging from 0.72 (2021) to 1 (2011 and 2014). The aggregated MIS was 0.08, ranging from 0.0 (2011 and 2014) to 0.28 (2021). The FAR was virtually 0, being always equal to 0.0, with the only exception being 2017, when it was 0.1 (Table 2).

**Table 2:** Capacity of the MIMESIS-2 model to correctly model laboratory-confirmed human WNV cases by year and in the whole observed time period.

Year	Inf Mun	$\sum {IH}_{OBS}$	POD^§^	FAR^§§^	MIS^§§§^	CSI^§§§§^
2010	38	262	0.9737	0	0.0263	0.9737
2011	46	100	1	0	0	1
2012	42	157	0.7381	0	0.2619	0.7381
2013	35	85	0.8857	0	0.1143	0.8857
2014	7	15	1	0	0	1
2015	0	0	–	–	–	–
2016	0	0	–	–	–	–
2017	10	48	0.9	0.1	0.1	0.8182
2018	85	306	0.9882	0	0.0118	0.9882
2019	56	223	0.9286	0	0.0714	0.9286
2020	48	144	0.9583	0	0.0417	0.9583
2021	18	58	0.7222	0	0.2778	0.7222
2010–2021	385	1398	0.9247	0.0028	0.0753	0.9223

Reported are the single evaluation criteria (POD, FAR, MIS, CSI) and the number of municipalities with at least one laboratory-confirmed human WNV case (Inf Mun) and the total number of laboratory-confirmed human WNV cases in Greece ($\sum {IH}_{OBS}$).

^§^$$\mathrm{POD}=\mathrm{Hits}/(\mathrm{Hits}+\mathrm{Misses})$$.

^§§^$$\mathrm{FAR}=(\mathrm{False Alarms})/(\mathrm{Hits}+\mathrm{False Alarms})$$.

^§§§^$$\mathrm{MIS}=\mathrm{ Misses}/(\mathrm{Hits}+\mathrm{Misses})$$.

^§§§§^$$\mathrm{CSI}=\mathrm{Hits}/(\mathrm{Hits}+\mathrm{False Alarms}+\mathrm{Misses})$$.

with Hits (municipalities with cases both observed and simulated for a specific year), False alarms (municipalities with cases simulated but not observed for a specific year), and Misses (municipalities with cases observed but not simulated for a specific year).

### Magnitude and timing of WNV events: performance and comparison with MIMESIS

To evaluate the ability of MIMESIS-2 to capture the magnitude and timing of WNV events, we first considered the discrepancy between the overall number of observed and modelled WNV cases during the 12-year period for each municipality. Out of the 153 municipalities where at least one case was modelled across the 12 years, 76 (50%) had at most 4 modelled cases of WNV: 1, 2, 3, and 4 WNV cases were modelled in 22, 31, 13, and 10 municipalities, respectively. In 42 municipalities, the number of modelled cases ranged from 5 to 10 (which, as for the observed WNV cases, coincided with the third quartile), and in 32 municipalities, the number ranged from 11 to 47, while the remaining 3 municipalities had 55, 70, and 99 modelled cases (Fig. 1).

**Figure 2:**
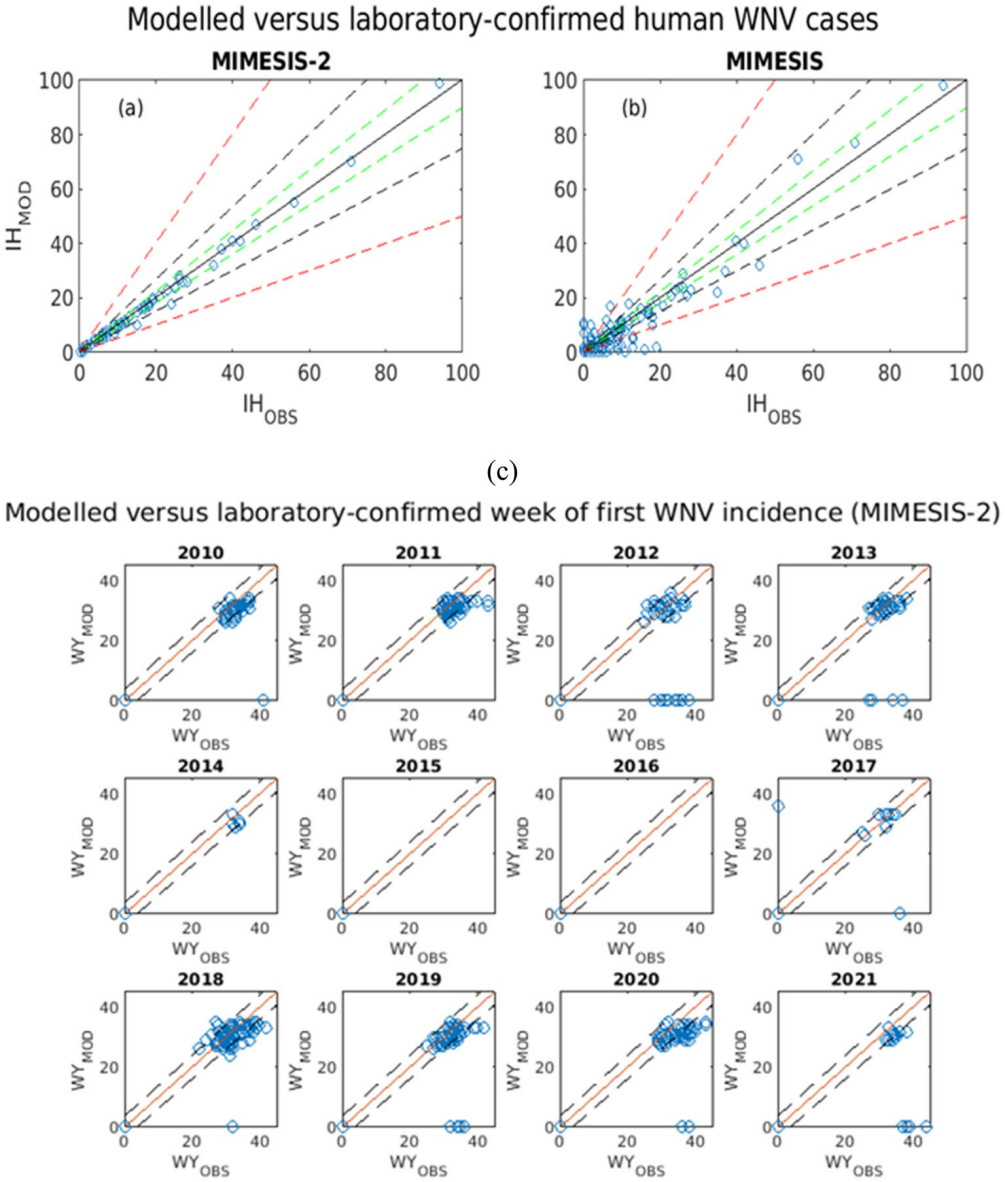
(**a**) For MIMESIS-2, modelled (*IH*_*MOD*_) vs. observed (*IH*_*OBS*_) human WNV cases in each municipality in the period 2010–2021. The inner black line represents the main diagonal where ideally the points would lie in case of perfect fit, while the dashed green, black and red lines represent, respectively, the ± 10%, ± 25% and ± 50% error margin. (**b**) Same quantities for MIMESIS. (c). Breakdown of the week of first WNV incidence by year. Plotted are the modelled quantities (WY_MOD_) for each of the 325 municipalities on the y-axis and the observed quantities (WY_OBS_) on the x-axis. The continuous line represents the main diagonal where ideally the points would lie in case of perfect fit, while the dashed lines represent the ± 4-week error margins.

The MIMESIS-2 model closely replicated the total number of laboratory-confirmed WNV cases during the 12-year period. When considering only the 154 municipalities that recorded at least one WNV event during the considered period (excluding the true negatives), for 140 of them, the modelled number of cases fell within a ± 10% error range of the observed value, whereas for 149 the modelled number of cases fell within the ± 25% error margin. Only two municipalities showed a percent error above 50%. These were particular instances where only one WNV case was reported throughout the considered period, while MIMESIS-2 fitted zero human cases. For the original MIMESIS model, 63 and 84 municipalities fell within the ± 10% and ± 25% error margins, respectively, while 31 municipalities—mainly those where few cases were observed— had a relative error ≥ 100% (Fig. 2).

To further evaluate the bias of the model across all municipalities and years, we explored the difference between the yearly modelled and observed human WNV cases both with MIMESIS-2 and the original MIMESIS (IH_MOD_-IH_OBS_) across municipalities. In MIMESIS-2, we excluded 3,514 true negative cases to avoid distorted conclusions. For the remaining 386 cases, the mean bias was -0.04 indicating a possibly unbiased model, with the standard deviation (SD) of the residuals equal to 0.66 (original MIMESIS: mean bias 0.33, SD 2.07, after removing 3,387 true negatives) (Supplementary Fig. 1).

Across the 325 municipalities and the 12 years, 385 WNV events were observed, while on 3,515 occasions, no laboratory-confirmed human WNV cases were reported; on 162 occurrences, 1 case was reported, and on 67 and 39 occasions, 2 and 3 cases were reported, respectively. The maximum yearly number of human WNV cases observed in a single municipality was 38. Considering the modelled human WNV cases with MIMESIS-2, the distribution of the 356 hits ranged between 1 and 37 modelled cases, closely mimicking the distribution of the observed cases, since 1, 2 and 3 human WNV cases were modelled on 129, 72 and 37 occasions, respectively. For the 29 misses, the observed numbers of human cases were 1 (24 times), 2 (3 times), or 3 (2 times). The only false alarm was produced in the Pellas municipality, where WNV events were observed in 8 out of the 12 years.

We evaluated the timing of the first occurrence of WNV in humans for any municipality and year. Ignoring the municipalities with zero cases, the observed and MIMESIS-2-modelled first WNV cases occurred between weeks 22 and 44 and weeks 24 and 36, respectively. Modelled values tended to be dispersed around the observed ones: excluding the 3514 true negatives, 290 (75.13%) of the remaining 386 cases fell into the ± 4-week error margins from the observed cases (Fig. 2). This translated into a much lower bias of the week of first appearance (WY_MOD_-WY_OBS_) with respect to MIMESIS (Supplementary Fig. 2).

### Case study: The Pellas municipality

In addition to presenting the overall performance of the model throughout different years and Greek municipalities, we highlight here the capacity of the model to capture population-specific behaviour and epidemiological features, such as the force of infection, that is, the rate at which susceptible humans, birds, and mosquitoes become infected, by presenting a single municipality case study for the municipality of Pellas. The Pellas municipality had the highest number of observed WNV cases over the 12-year period with a total of 94 human WNV cases, 38 in 2010, 16 in 2018, and 13 in 2021, no cases from 2014 to 2017, and between 4 to 8 cases in the remaining years.

**Figure 3:**
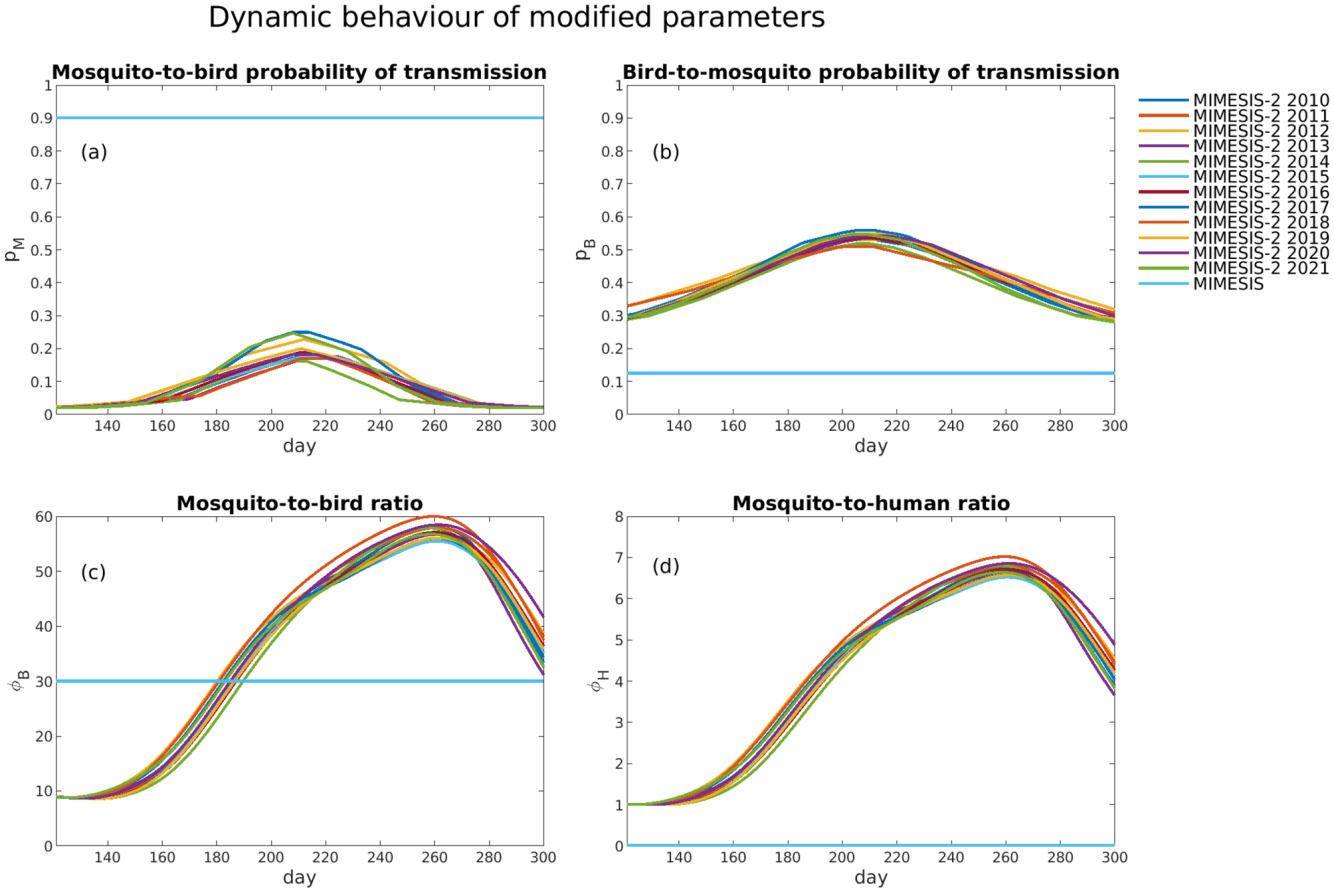
The temporal evolution during May to October of the (**a**) mosquito-to-bird probability of transmission, ${p}_{M}$, (**b**) bird-to-mosquito probability of transmission, ${p}_{B},$ (**c**) mosquito-to-bird ratio, ${\varphi }_{B},$ and (**d**) mosquito-to-human ratio, ${\varphi }_{H},$ for both MIMESIS-2 across different years and MIMESIS for each of the 12 years from 2010 to 2021. The plots refer to the simulations for the municipality of Pella.

We considered the impact arising from the changes in parameters defining the forces of infection. In addition to the introduction of bird (${\psi }_{B}$) and human (${\psi }_{H}$) host selections, changes included modifications for the mosquito-to-bird (${p}_{M}$) and bird-to-mosquito (${p}_{B}$) probabilities of transmission, whose values were made temperature-dependent following Vogels et al.^21^, and the replacement of the mosquito-to-bird (${\varphi }_{B}$) and mosquito-to-human (${\varphi }_{H}$) ratios with their dynamic counterparts, ${N}_{M}/{N}_{B}$ and ${N}_{M}/{N}_{H}$, respectively (Fig. 3). We used the May–October period for the 12 years that were considered, because this is the part of the year when *Culex pipiens* mosquitoes are reproductively active and the majority of human WNV cases are reported. In each year of the 12-year period, ${p}_{M}$ started from 0.02, reached its peak—ranging from 0.16 to 0.25—in midsummer, and then decreased to the initial values (in the original model, ${p}_{M}=0.9$). Similarly, ${p}_{B}$ started from 0.28, peaked in the same time interval—with maximal values ranging from 0.51 to 0.56—and then returned to the initial values (in the original model, ${p}_{B}=0.125$). Additionally, the dynamic specifications of ${\varphi }_{B}$ and ${\varphi }_{H}$ were shown to play an important role. Whereas in MIMESIS ${\varphi }_{B}=30$, in MIMESIS-2 the values started at approximately 8.6 and peaked in late summer when more human WNV cases are reported, reaching values of approximately 57, before decreasing to values ranging from 31.15 to 41.60 in late October. In MIMESIS, ${\varphi }_{H}$ was calibrated at the municipality level, and for Pellas municipality, it was 0.0001, whereas the dynamic counterpart in MIMESIS-2 showed a temporal evolution with a shape (but different scale) similar to that of ${\varphi }_{B}$, starting from values of approximately 1, peaking in late summer to values of approximately 7, and then decreasing to values of approximately 4 in late October.

**Figure 4:**
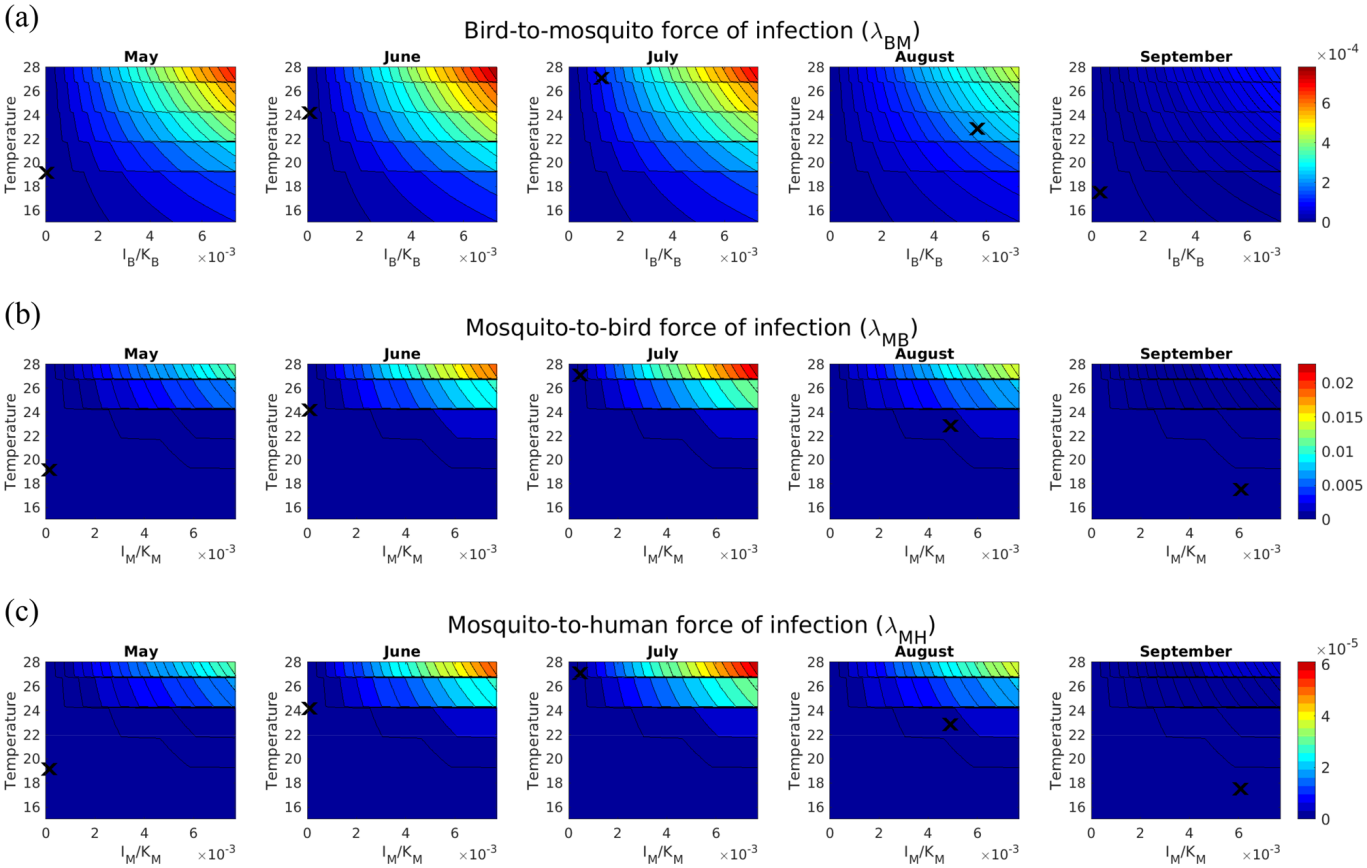
Contour plots of the forces of infection for May to September for different values of the relative abundance of infected hosts/vectors with respect to the carrying capacity and the temperature. All the other quantities were fixed to the amounts obtained in the simulations for Pellas municipality for 2021 at the end of the corresponding month. (**a**) Bird-to-mosquito force of infection (${\lambda }_{BM}$) as a function of the relative abundance of infected birds (${I}_{B}$) with respect to the bird carrying capacity (${K}_{B}$) and temperature. (**b**) Mosquito-to-bird force of infection (${\lambda }_{MB}$) as a function of the relative abundance of infected mosquitoes (${I}_{M}$) with respect to the mosquito carrying capacity (${K}_{M}$) and temperature. (**c**) Mosquito-to-human force of infection (${\lambda }_{MH}$) as a function of the relative abundance of infected mosquitoes (${I}_{M}$) with respect to the mosquito carrying capacity (${K}_{M}$) and temperature. The ranges for ${I}_{B}/{K}_{B}$ and ${I}_{M}/{K}_{M}$ were fixed, increasing the maximum modelled value by 20% for the considered period, while the range for the temperature was chosen considering that in the period of interest, the average daily temperature ranged from 16.6 to 27.1 degrees Celsius. Black crosses represent the modelled values for 2021.

Changes in these parameters enter into the expression for the forces of infection. It is of major practical interest to investigate how the values for the forces of infection resulting from MIMESIS-2 may vary for different values of the relative abundance of the vectors with respect to the corresponding carrying capacity and the temperature in different months (Fig. 4). As expected, all forces of infection increased with both the temperature and the relative abundance of the infectious vertebrate hosts. It is worth noting the importance of day length, as this affects the fraction of nondiapausing mosquitoes, ${\delta }_{M}$, and causes the forces of infection, all other things being equal, to be potentially higher in June and July than in the other months. However, in these two months, the modelled forces of infection tend to be smaller than those in August due to the lower abundance of infectious hosts.

The bird-to-mosquito force of infection, ${\uplambda }_{BM}$, took values on the order of 10^–4^, with possible peaks of approximately 7 × 10^–4^ in the case of high temperature and high prevalence of birds in June and July, which were nevertheless not reached due to a low abundance of infected birds in that period. Considering the months of July and August 2021 for illustrative purposes, the resulting modelled values were 1.20 × 10^–4^ and 2.19 × 10^–4^, respectively, with the increase in August explained by a higher abundance of infected birds in that period. It is worth noting that if the infection across birds had a lead period of two weeks, the resulting ${\uplambda }_{BM}$ in July would become 3.91 × 10^–4^ (+ 226%), while an increase in the average temperature in August by 1 °C would result in ${\uplambda }_{BM}$= 2.36 × 10^–4^ (+ 8%). The mosquito-to-bird, ${\uplambda }_{MB}$, and mosquito-to-human, ${\uplambda }_{MH}$, forces of infection showed similar qualitative behaviours, albeit at different scales, and in this case, they were higher in August due to a higher prevalence of infected *Culex* mosquitoes in that month. More specifically, ${\uplambda }_{MB}$ equalled 1.06 × 10^–3^ and 1.22 × 10^–3^ at the end of July and August, respectively, and an expected two weeks for the infection of mosquitoes would result in ${\uplambda }_{MB}$=4.15 × 10^–3^ (+ 292%) at the end of July, while an increase in the average temperature in August by 1 °C would result in ${\uplambda }_{MB}$= 1.31 × 10^–3^ (+ 7%) at the end of August. Finally, ${\uplambda }_{MH}$=2.86 × 10^–6^ at the end of July, while ${\uplambda }_{MH}$=3.26 × 10^–6^ at the end of August, with the anticipation of the infection among mosquitoes by two weeks resulting in ${\uplambda }_{MH}$=1.12 × 10^–5^ (+ 290%) and an increase in the average August temperature by 1 °C leading to ${\uplambda }_{MH}$=3.51 × 10^–6^ (+ 8%). It is worth recalling that since we calibrated the model on the number of reported laboratory-confirmed human WNV cases, ${\uplambda }_{MH}$ represents the rate at which susceptible humans contract infection and become symptomatic leading to a recorded human WNV case.

**Figure 5:**
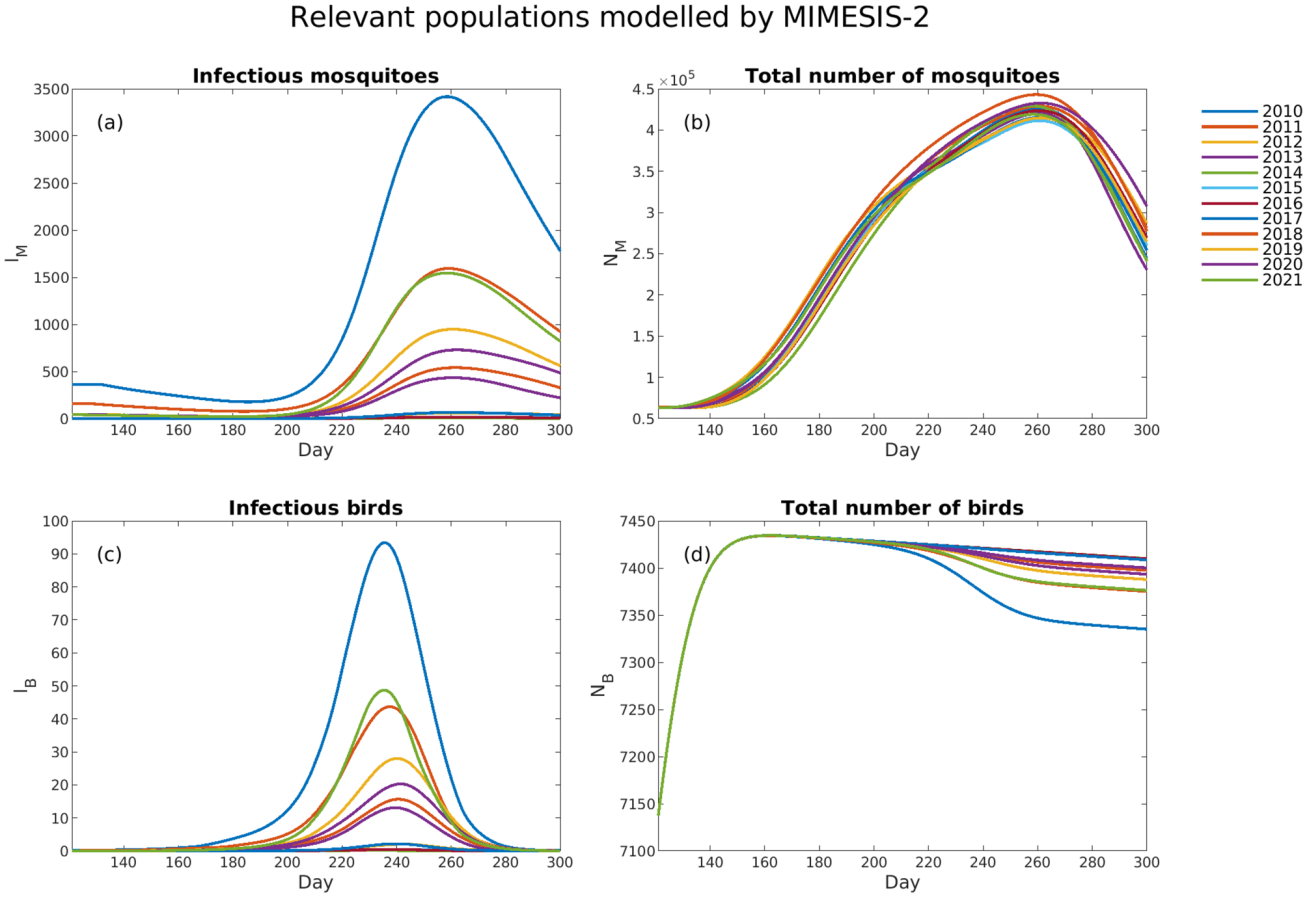
The temporal evolution during May to October of (**a**) the number of infected mosquitoes modelled by MIMESIS-2 (${I}_{M}$), (**b**) the total number of mosquitoes modelled by MIMESIS-2 (${N}_{M}$)$,$ (**c**) the number of infected birds modelled by MIMESIS-2 (${I}_{B}$)$,$ and (**d**) the total number of birds modelled by MIMESIS-2 (${N}_{B}$) for each of the 12 years from 2010 to 2021. The plots refer to the simulations for the municipality of Pella.

**Figure 6:**
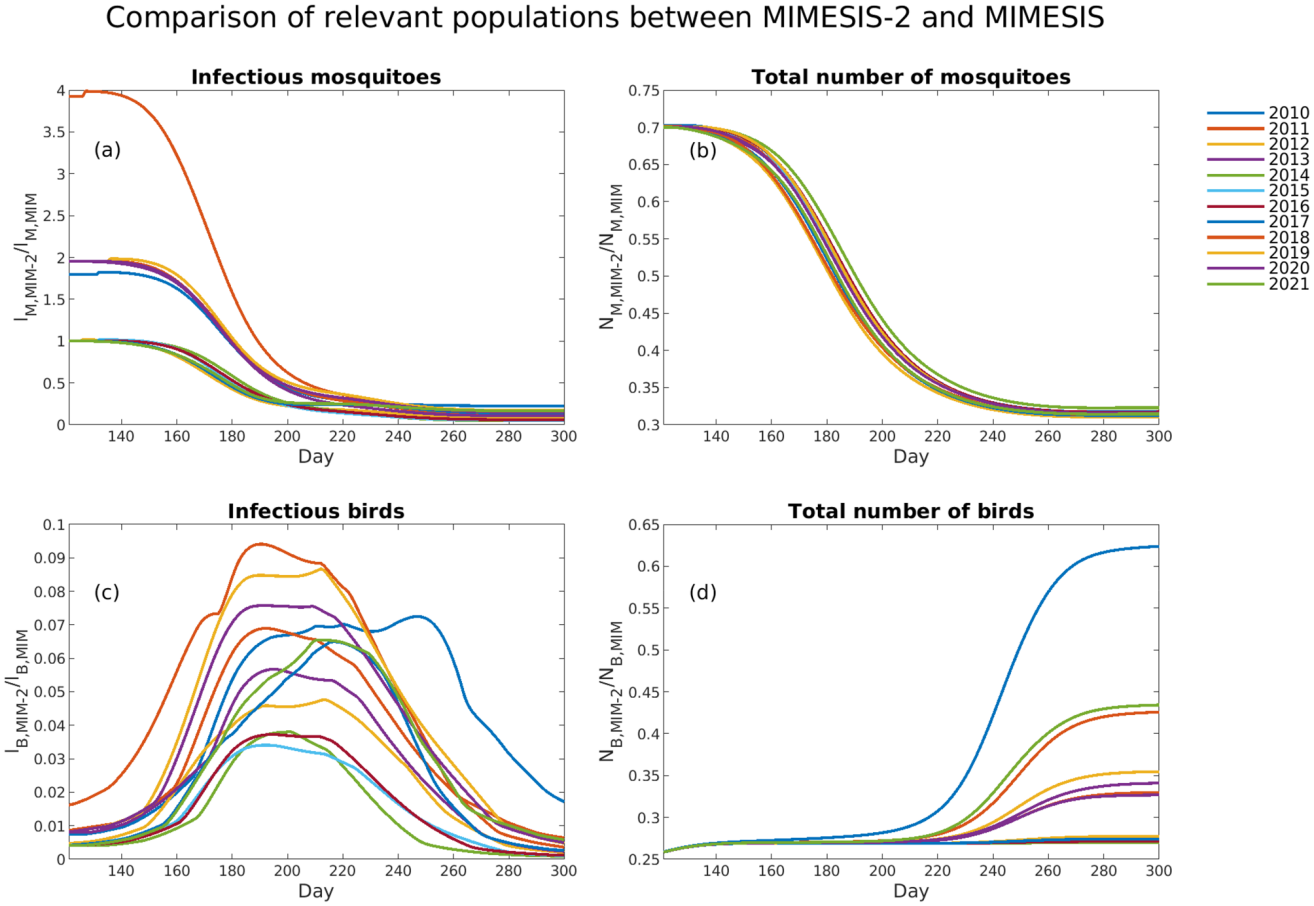
The temporal evolution during May to October of (**a**) the ratio between the number of WNV-infected mosquitoes modelled by MIMESIS-2 (${I}_{M,MIM-2}$) and the ratio modelled by MIMESIS (${I}_{M,MIM}$), (**b**) the ratio between the total number of mosquitoes modelled by MIMESIS-2 (${N}_{M,MIM-2}$) and the ratio modelled by MIMESIS (${N}_{M,MIM}$)$,$ (**c**) the ratio between the number of infected birds modelled by MIMESIS-2 (${I}_{B,MIM-2}$) and the ratio modelled by MIMESIS (${I}_{B,MIM}$)$,$ and (**d**) the ratio between the total number of birds modelled by MIMESIS-2 (${N}_{B,MIM-2}$) and the ratio modelled by MIMESIS (${N}_{B,MIM}$) for each of the years from 2010 to 2021. The plots refer to the simulations for the municipality of Pella.

We explored changes in the populations of infectious hosts and the total population number for both mosquitoes and birds over 2010–2021 for the period spanning from May to October (Figs. 5 and 6). The population of infected mosquitoes (${I}_{M}$) was initialised by calibration (see the Methods section). Each year, after a short period in which the population of infected mosquitoes slightly decreased due to a very small number of infectious birds (${I}_{B}$) that prevented the infection from spreading, it started growing substantially during summer, reaching its peak in late summer, coinciding with the period when most human cases were recorded. The observed increase in ${I}_{M}$ was combined with the growth ${I}_{B}$ at approximately the same time (with a slightly anticipated peak), which had an amplification effect on the spread of the infection. Both ${I}_{M}$ and ${I}_{B}$ showed significant yearly variation, with higher modelled numbers in years where more human WNV cases were reported. The modelled total population of mosquitoes (${N}_{M}$) did not show significant interannual variability, always peaking in late summer. Finally, the overall population of birds (${N}_{B}$) did not show any variability in the first part of the year, when an increase due to immigration and offspring generation was observed, whereas it had a moderate interannual variability in the second half of the year. These differences may be due to heterogeneous numbers of observed infected, dead and immune birds.

Comparison of these population dynamics with those of MIMESIS revealed interesting patterns (Fig. 6). Considering the relative number of mosquitoes in MIMESIS-2 with respect to MIMESIS, the populations in MIMESIS tended to grow faster due to a higher mosquito carrying capacity (${K}_{M}$) in the original model (${K}_{M}$ ≈ 8.3 × 10^5^ in MIMESIS versus ${K}_{M}$ ≈ 2.4 × 10^5^ in MIMESIS-2), resulting in a decrease in the ratio between the amounts modelled by MIMESIS-2 and the ones modelled by MIMESIS. Significant interannual variability could be seen in the first part of the year for infectious mosquitoes, where different initial calibration values played an important role. For the populations of birds, until midsummer, the overall number modelled by MIMESIS-2 tended to be approximately 1/4 that of MIMESIS, while as of July, different patterns were observed due to the higher mortality of birds in the original MIMESIS model. In years with higher virus spread, higher mortality was reflected in a sharper decrease in bird populations; therefore, the ratio between the population modelled by MIMESIS-2 and that modelled by MIMESIS increased up to approximately 0.6 (2010).

## Discussion

We extended a previous climate-dependent epidemiological model for WNV^26^ by including vector-specific characteristics of WNV transmission, such as mosquito host selection and temperature-dependent virus transmission. The aim was to better quantify the emergence of WNV events and the week of the first occurrence of a human WNV case. MIMESIS-2 showed improved performance in comparison to the original MIMESIS model^26^ in this respect. Moreover, it showed improved performance indices on the aggregated level and at geographical and temporal breakdowns.

When considering the 154 municipalities that reported human WNV cases over the period 2010–2021, both the POD and MIS were above 0.8 in 139 (90%) of them, whereas the MIS was below 0.2 in 142 (92%) municipalities. For the municipalities with modelled human WNV cases (153), the FAR was always below 0.2, with only one false alarm produced. These results were consistent with the multiannual evolution of the indices. The POD and MIS ranged from 0.72 (2021) to 1.0 (2011 and 2014), with aggregate levels across years equal to 0.92. The MIS ranged from 0.0 (2011 and 2014) to 0.28 (2021), with an aggregate value of 0.08. The FAR was always zero, except in 2017, when it was equal to 0.1. These high POD and CSI values, combined with the low FAR and MIS values, showed that the model has enough flexibility to capture different patterns observed in time and space.

MIMESIS-2 showed improved performance both in terms of capturing epidemiological patterns in years and municipalities where few cases were observed, as well as in terms of a significantly reduced number of false alarms with only one produced over the 12-year period. Moreover, MIMESIS-2 showed negligible bias in modelling the observed laboratory-confirmed human WNV cases and an improved capacity to model both high and low numbers of cases, unlike MIMESIS, in which a tendency towards underestimation was observed.

The model showed the capacity to correctly capture the week of the first occurrence of a human WNV case. Excluding the correct negatives, the week of the first human case modelled by MIMESIS-2 fell within the ± 4-week bands from the observed value in 75% of cases, while this percentage dropped to 44% for MIMESIS. This supports the notion of using the model as a predictive tool, although the fitted values showed a slightly smaller dispersion than the observed values, seemingly due to some difficulties in capturing late-case occurrence.

MIMESIS-2 allows for the exploration of important features of virus transmission, such as the forces of infection of birds, mosquitoes, and humans and their dependence on host abundance and temperature, which can be used to develop efficient strategies to lower the risk of epidemics. The Pella case study showed the important role of characteristics such as bird (${\psi }_{B}$) and human (${\psi }_{H}$) host selection, as well as the mosquito-to-bird (${p}_{M}$) and bird-to-mosquito (${p}_{B}$) probability of transmission. The study of the infectious and overall numbers of mosquitoes and birds showed that, although the dynamics had similar qualitative behaviours, the resulting numbers across the two models could be substantially different. This points to further research directions that focus on the validation of the fraction of infectious mosquitoes and amplifying host birds (e.g., the role of avian host competence and flock immunity^28^) and the study of possible changes in mosquitoe late summer feeding patterns.

Future research could focus on the feeding behaviours (patterns and preferences) of mosquitoes in relation to the availability of potential hosts in the immediate environment. Disentangling this relationship could help define virus transmission dynamics and the best timing for specific interventions (e.g., larvicide as a preventive approach or adulticide as a reaction to viral amplification). The opportunistic nature of WNV-transmitting mosquitoes and the scale of influence of the relationship between mosquitoes and hosts might be a challenge to the implementation of such models, especially for predictive aims^29^. For this reason, the generalization of these results to larger areas such as regions or states still appears complex. Conversely, acknowledging the role of different potential host populations could help disentangle, for instance, which among the different avian species can act as amplifying hosts in the cycle of WNV. Several avian species do not develop sufficient viremia to infect mosquitoes; this aspect can be modelled alongside host-specific (e.g., nesting season) and other ecological aspects (e.g., temperature and precipitation) to explore the eventual association of specific host species with human cases as well as with the proportion of WNV-infected mosquitoes. The impact of environmental conditions on the intrayear (e.g., changes in larval mortality associated with after-rain pond evolution) and multiyear (e.g., overwintering) variability in the mosquito population will allow a detailed validation of entomological subpopulations in a broader time frame.

Finally, the role of infectious birds as virus reservoirs is certainly important. In our results, the number of infectious mosquitoes started to grow considerably when enough infectious birds were present in the environment, making virus circulation much more effective. We assumed that the infection always started with mosquitoes, but future research could study different scenarios with the infection starting from birds that may keep the infection over winter (e.g., due to chronic infection) or introduce the virus during spring migration. Although challenging in practice, estimating the fraction of infectious birds would be a key aspect to study, both to better understand how virus transmission can survive during winter and to better understand transmission dynamics across different years.

Our results show that epidemiological models can be used to describe host-specific mosquito dynamics. In turn, these dynamics could help develop new sets of more region-specific interventions (such as e.g., specific time frames and campaigns to control major amplifying hosts).

We included a general assessment of host selection. We modified the original MIMESIS model^26^ to force a proportion of mosquito bites to be assigned to birds or mammals. In addition, we considered that a proportion of bites that would originally target birds would not re-enter the cycle, accounting in this way for the unknown proportion of avian species that represent a noncompetent WNV host. The proportion we considered for this study was taken from the study on feeding patterns of *Culex pipiens* and *Culex restuans* by Hamer et al.^30^. It is important to note that this study presented controversies. On the one hand, the proportion of observed blood meals taken from humans was higher than in other studies on *Culex pipiens* and *Culex restuans*^31^. This was considered a result of not accounting for the proportion of *Culex pipiens* (mainly ornithophilic), *Culex molestus* (mainly mammophilic), and hybrids in the mosquito population^31^. Moreover, this study considered only two potential WNV-transmitting mosquitoes with specific population dynamics in addition to their host preferences (e.g., population peak time) in a very specific setting, such as an urban environment in North America. To concretely include host selection dynamics in an epidemiological model such as MIMESIS-2, more information on the observed area (e.g., main vector population) is needed. Lacking these data in the current approach, the broader values for the vector host selection could represent a general means that accounts for the different feeding patterns of the various WNV-transmitting *Culex* mosquitoes, as well as for the different potential environments (e.g., urban, rural, and semirural, which might represent a critical aspect, as the potential overall host population is greater in rural areas, whereas the proportion of competent hosts is higher in urban areas).

Furthermore, we aimed to include a representation of the effect of temperature on the ability of WNV-transmitting mosquitoes to be infected and to transmit WNV. Broadly following Vogels et al.^21^, we oriented this study around three temperature points: 18, 23, and 28 °C. When interpreting this approach, it is important to consider that these temperatures were obtained in experimental settings, whereas in real-world settings, it is highly unlikely that the mosquitoes would be exposed to constant temperatures, even if this might not represent a capital issue^32^. For this reason, we considered five temperature intervals obtained by adding two fictional observations at 20.5 and 25.5 °C, where the values of the infection and transmission probabilities were set equal to the mean value reported for the two adjacent temperature points reported in Vogels et al.^21^. We then divided the temperature values according to five intervals delimited by the midpoints of 18, 20.5, 23, 25.5 and 28 °C and took constant values for the infection and transmission probabilities for these intervals. With this approach, we expected to better mimic real-world temperature oscillations. Moreover, in Vogels et al.^21^, there was no mention of the impact on WNV infection and transmission for temperatures exceeding the aforementioned values. Nevertheless, the inclusion of additional flexibility to represent the impact of temperatures on the mosquito-specific ability to spread WNV might need further knowledge to be included in a more precise way.

Our results indicate that mosquito-specific characteristics such as host selection and temperature-dependent virus transmission included in a climate-dependent epidemiological model for WNV infections in humans increase the model’s potential to model the occurrence of human WNV cases. MIMESIS-2 modelled human WNV cases in Greece with enhanced performance when compared to the original MIMESIS model. However, the updates that we introduced present limitations based on the actual lack of data on mosquito-specific characteristics both in general and in specific settings (Greece). Epidemiological models have been used to understand mosquito host dynamics, but they have to be informed from proper field epidemiological studies that are currently lacking.

## Methods

### Epidemiological model

The model in this study, which we called MIMESIS-2, is an extension of the climate-dependent spatial epidemiological model MIMESIS (spatial dynaMIcal Model for wESt nIle viruS) by Angelou et al.^26^.

MIMESIS was developed to model the populations of all the species involved in the WNV transmission cycle (mosquitoes, birds, and humans) in the 325 municipalities of Greece between 2010 and 2019, downscaling to the municipality level of the previous model for WNV by Kioutsioukis & Stilianakis^25^, where a sensitivity analysis also was carried out for the Regional Unit of Thessaloniki in North Greece.

MIMESIS incorporated epidemiological and meteorological data, as well as demographic and geographic information about the municipalities. Epidemiological data on laboratory-confirmed cases of WNV infections in humans were obtained from the Hellenic Centre for Disease Control and Prevention (HCDCP). Meteorological data (mean, maximum and minimum daily temperatures) were obtained from ERA 5 of the European Centre for Medium-Range Weather Forecasts (ECMWF). Before being included in the model, temperature data were smoothed to eliminate fluctuations with a Kolmogorov-Zurbenko filter.

MIMESIS presented 14 compartments, which were retained in the updated MIMESIS-2: 4 for the *Culex* mosquitoes (nonadult mosquitoes [${L}_{M}$], susceptible adult mosquitoes [${S}_{M}$], exposed adult mosquitoes [${E}_{M}$], and infectious adult mosquitoes [${I}_{M}$]) and 5 each for birds and humans (susceptible birds [${S}_{B}$], susceptible humans [${S}_{H}$], exposed birds [${E}_{B}$], exposed humans [${E}_{H}$], infectious birds [${I}_{B}$], infectious humans [${I}_{H}$], recovered/resistant birds [${R}_{B}$], recovered/resistant humans [${R}_{H}$], dead birds [${D}_{B}$], and dead humans [${D}_{H}$]). Recovered birds and humans were presumed immune to infection due to acquired immunity. In the original MIMESIS model, the forces of infection (${\uplambda }_{BM}, {\uplambda }_{MB}, {\uplambda }_{MH}$) were defined as:

$${\uplambda }_{BM}\left(T\right)={\delta }_{M}k\left(T\right){p}_{B}\frac{{I}_{B}}{{K}_{B}}$$

$${\uplambda }_{MB}\left(T\right)={\delta }_{M}k\left(T\right){p}_{M}{\varphi }_{B}\frac{{I}_{M}}{{K}_{M}}$$

$${\uplambda }_{MH}\left(T\right)={\delta }_{M}k\left(T\right){p}_{M}{\varphi }_{H}\frac{{I}_{M}}{{K}_{M}}$$

Below are all the changes made to these expressions to improve the original model. The complete specifications of the updated MIMESIS-2 model are available in the Supplementary Information.

### Changes to the forces of infection to include mosquito-specific aspects

#### Host selection

Following Hamer et al.^30^, we considered mosquitoes to have a higher preference for bird hosts than mammals. We controlled host selection, including two terms for the force of infections: bird host selection ${\psi }_{B}=0.7$ and human host selection ${\psi }_{H}=0.016$. The value for human host selection was the result of the calibration of our model on the number of reported laboratory-confirmed human WNV cases. Thus, the preference of mosquitoes to feed on humans (0.16) was multiplied by the estimated percentage of reported cases among all infected humans (0.10) to account only for the fraction of bites in a recorded human WNV case. The two terms ${\psi }_{B}$ and ${\psi }_{H}$ did not cover the whole range of potential bites given. This was chosen to account for the bites given to birds and mammals that are not able to develop sufficient viremia to reinfect mosquitoes and thus do not take an amplifying role in the cycle.

#### Dynamic population ratio

The original MIMESIS model controlled the ratio between the population of mosquitoes and the populations of birds and humans via the constant terms (${\varphi }_{B}$ and ${\varphi }_{H}$ for birds and humans, respectively). In the updated version, we allowed for the mosquito-to-bird ratio and mosquito-to-human ratio to change dynamically over time, replacing them with the ratio between the population of mosquitoes ${N}_{M}$ and the populations of birds ${N}_{B}$ and humans ${N}_{H}$, that is, ${\varphi }_{B}= \frac{{N}_{M}}{{N}_{B}}$ and ${\varphi }_{H}= \frac{{N}_{M}}{{N}_{H}}$.

#### Temperature-dependent transmission

Broadly following Vogels et al.^21^, the probabilities of transmission (mosquito-to-bird [${p}_{M}$] and bird-to-mosquito [${p}_{B}$]) were corrected in their values to be temperature dependent. We divided the temperature range into five intervals (in degrees Celsius) and considered constant values of ${p}_{M}$ and ${p}_{B}$ for such intervals: $\left(-\infty , 19.25\right], \left(19.25, 21.75\right], \left(21.75, 24.25\right], \left(24.25, 26.75\right],\mathrm{ and }(26.75, +\infty )$. Then, for the first, third and fifth intervals, we took the values of ${p}_{M}$ and ${p}_{B}$ reported in Vogels et al.^17^ for the temperature values 18, 23, and 28 °C. For the second and fourth intervals, we took the average values between the two adjacent intervals. Thus, the resulting values for ${p}_{M}\left(T\right)$ and ${p}_{B}\left(T\right)$ were set as follows:

$${p}_{M}\left(T\right)=\left\{\begin{array}{c}0.02 \,\, \, if \,\, T\le 19.25^\circ{\rm C} \\ 0.04 \,\, \, if \,\,19.25<T\le21.75^\circ{\rm C} \\ 0.06 \,\, \, if \,\, 21.75<T\le24.25^\circ{\rm C} \\ 0.20 \,\, \, if \,\, 24.25<T\le26.75^\circ{\rm C} \\ 0.34 \,\, \, if T>26.75^\circ{\rm C} \end{array}\right.$$

and

$${p}_{B}\left(T\right)=\left\{\begin{array}{c}0.28 \,\, \, if \,\, T\le 19.25^\circ{\rm C} \\ 0.39 \,\, \, if \,\, 19.25<T\le21.75^\circ{\rm C} \\ 0.50 \,\, \, if \,\,21.75<T\le24.25^\circ{\rm C} \\ 0.56 \,\, \, if \,\, 24.25<T\le26.75^\circ{\rm C} \\ 0.62 \,\, \, if \,\, T>26.75^\circ{\rm C} \end{array}\right.$$

The resulting values were then smoothed with a moving average to eliminate fluctuations.

#### Final expression for the forces of infection

After the above changes were implemented, the resulting forces of infection were as follows (with the changes reported in bold):

$${\lambda }_{BM}\left(T\right)={\delta }_{M}k\left(T\right){{\varvec{\psi}}}_{{\varvec{B}}}{{\varvec{p}}}_{{\varvec{B}}}\left({\varvec{T}}\right)\frac{{I}_{B}}{{K}_{B}}$$

$${\lambda }_{MB}\left(T\right)={\delta }_{M}k(T){{{\varvec{\psi}}}_{{\varvec{B}}}{\varvec{p}}}_{{\varvec{M}}}({\varvec{T}})\frac{{{\varvec{N}}}_{{\varvec{M}}}}{{{\varvec{N}}}_{{\varvec{B}}}} \frac{{I}_{M}}{{K}_{M}}$$

$${\lambda }_{MH}\left(T\right)={\delta }_{M}k(T){{{\varvec{\psi}}}_{{\varvec{H}}}{\varvec{p}}}_{{\varvec{M}}}({\varvec{T}})\frac{{{\varvec{N}}}_{{\varvec{M}}}}{{{\varvec{N}}}_{{\varvec{H}}}} \frac{{I}_{M}}{{K}_{M}}$$

#### Further changes

Additional changes to MIMESIS considered WNV-induced bird mortality (ν_B_), which was changed from the original *ν*_*B*_ = 0.7 to *ν*_*B*_ = 0.05 to represent the lower bird mortality observed in Europe compared to the United States.

In the MIMESIS model, the maximum number of birds per municipality, i.e., the carrying capacity (${K}_{B}$) was equal to the initial population of susceptible birds, that is, ${K}_{B}={S}_{B,0}$, with ${S}_{B,0}$ being the initial number of susceptible birds (which was also the initial total number of birds). In MIMESIS-2, we set ${K}_{B}={1.2*S}_{B,0}$ so that we allowed ${K}_{B}$ to be 20% more than the initial population of birds. This is because we expected to observe an increase in the overall population of birds during spring (e.g., migration and birth of offspring). Conversely, the carrying capacity of mosquitoes (${K}_{M}$) was set broadly following the MIMESIS model as ${K}_{M}=30*{K}_{B}$.

In addition to the aforementioned formal updates to the MIMESIS model, we also included the years 2020 and 2021 in MIMESIS-2.

#### Model calibration

We calibrated our MIMESIS-2 model based on the mean square error (MSE) between the modelled number of human WNV cases and the observed number across all 325 municipalities and 12 years. The compartments involved in the calibration were the initial numbers of susceptible mosquitoes (${S}_{M, 0}$), susceptible birds (${S}_{B, 0}$) and infected mosquitoes (${I}_{M, 0}$).

On the other hand, the initial number of susceptible humans (${S}_{H, 0}$) for each municipality was retrieved from the Hellenic Statistical Authority (HSA), while the initial values of mosquito larvae (${L}_{M, 0}$), exposed mosquitoes, birds and humans (${E}_{M, 0},{ E}_{B, 0}, {E}_{H, 0}$), infected birds and humans (${{I}_{B, 0}, I}_{H, 0}$), recovered birds and humans (${{R}_{B, 0}, R}_{H, 0}$) and dead birds and humans (${{D}_{B, 0}, D}_{H, 0}$) were set to zero. In detail, for the calibration process, the initial number of susceptible mosquitoes (${S}_{M, 0}$) for all 12 years in each municipality was set equal to the minimum number of nonhibernating mosquitoes ${N}_{M, min}$ in that municipality, which was allowed to take 10 possible values. For each municipality $m$, calling ${A}_{m}$ the area (in square kilometres) of the municipality, we considered as possible values ${N}_{M, min}\left(m\right)= \frac{\mathrm{50,000}}{\mathrm{3,680}}*{A}_{m}*c$, where $c$ ranges from 1 to 10 with a step of 1. To reduce possible overfitting issues, the rescaling factor $c$ was not municipality dependent but common across all municipalities. For the initial population of susceptible birds (${S}_{B, 0}$), calling ${b}_{d}(m)$ the density of birds in municipality $m$ per square kilometre, we calibrated ${S}_{B, 0}(m)$ (municipality dependent), making it equal to ${A}_{m}*{b}_{d}\left(m\right)$, with ${b}_{d}\left(m\right)$ ranging from 5 to 50 with a step of 5 (10 values). Finally, the initial number of infected mosquitoes (${I}_{M, 0}$) was calibrated for each municipality and each year (municipality and year dependent) by taking 10 possible values ranging from 1 to 401 with a step of 40. All calculations were conducted using MATLAB version 9.7 (R2019b) (*Natick, Massachusetts: The MathWorks Inc [2019]*) with a time step of one day.

#### Model verification and comparison

The developed MIMESIS-2 model was tested according to the same metrics as the original MIMESIS model. The performance indices were built starting from the following four quantities:

- the hits (municipalities with cases both observed and simulated for a specific year),
- the false alarms (municipalities with cases simulated but not observed for a specific year),
- the misses (municipalities with cases observed but not simulated for a specific year), and
- the correct negatives (municipalities with cases not observed or simulated for a specific year).

From these four quantities, we calculated the four performance indices used to evaluate the performance of the model:

- the probability of detection (POD), such as $\mathrm{POD}=\frac{\mathrm{Hits}}{\mathrm{Hits}+\mathrm{Misses}}$
- the miss rate (MIS), such as $\mathrm{MIS}= \frac{\mathrm{Misses}}{\mathrm{Hits}+\mathrm{Misses}}$
- the false alarm rate (FAR), such as $\mathrm{FAR}=\frac{\mathrm{False Alarms}}{\mathrm{Hits}+\mathrm{False Alarms}}$
- the critical success index (CSI), such as $\mathrm{CSI}=\frac{\mathrm{Hits}}{\mathrm{Hits}+\mathrm{False Alarms}+\mathrm{Misses}}$

All these parameters ranged from 0 to 1, with the best score being 1 for the POD and CSI and 0 for the MIS and FAR.

## Supplementary Information

Supplementary Information.

## Data Availability

The datasets generated during and/or analysed during the current study are available from the corresponding author upon reasonable request.
