# Supplementary material for: An epidemiological model for mosquito host selection and temperature-dependent transmission of West Nile virus
Source: Sci Rep. 2022 Nov 19;12:19946. doi: 10.1038/s41598-022-24527-5 (PMC9675847; doi:10.1038/s41598-022-24527-5)
Supplement: Supplementary file 1 — Supplementary Information. [file 41598_2022_24527_MOESM1_ESM.pdf]

Supplementary Information

The model

The equations specifying the evolution of the compartments of mosquitoes, birds and humans are as follows:

$$\frac{dL_M}{dt} = (b_L(T)\delta_M N_M - m_L(T)L_M)\left(1 - \frac{L_M}{K_M}\right) - b_M(T)L_M$$

$$\frac{dS_M}{dt} = -\lambda_{BM}(T)S_M + b_M(T)L_M - m_M(T)S_M$$

$$\frac{dE_M}{dt} = \lambda_{BM}(T)S_M - \gamma_M(T)E_M - m_M(T)E_M$$

$$\frac{dI_M}{dt} = \gamma_M(T)E_M - m_M(T)I_M$$

$$\frac{dS_B}{dt} = (b_b - (b_b - m_b)\frac{N_B}{K_B})N_B - \lambda_{MB}(T)S_B - m_B(T)S_B$$

$$\frac{dE_B}{dt} = \lambda_{MB}(T)S_B - \gamma_B E_B - m_B(T)E_B$$

$$\frac{dI_B}{dt} = \gamma_B E_B - \alpha_B I_B - m_B I_B$$

$$\frac{dR_B}{dt} = (1 - \nu_B)\alpha_B I_B - m_B R_B$$

$$\frac{dD_B}{dt} = \nu_B \alpha_B I_B$$

$$\frac{dS_H}{dt} = r_H N_H - \lambda_{MH}(T)S_H$$

$$\frac{dE_H}{dt} = \lambda_{MH}(T)S_H - \gamma_H E_H$$

$$\frac{dI_H}{dt} = \gamma_H E_H - \alpha_H I_H$$

$$\frac{dR_H}{dt} = (1 - \nu_H)\alpha_H I_H$$

$$\frac{dD_H}{dt} = \nu_H \alpha_H I_H$$

with forces of infection equal to

$$\lambda_{BM}(T) = \delta_M k(T)\psi_B(T)p_B(T)\frac{I_B}{K_B}$$

$$\lambda_{MB}(T) = \delta_M k(T)\psi_B(T)p_M(T)\frac{N_M}{N_B}\cdot\frac{I_M}{K_M}$$

$$\lambda_{MH}(T) = \delta_M k(T)\psi_H(T)p_M(T)\frac{N_M}{N_H}\cdot\frac{I_M}{K_M}$$

Supplementary Tables

Supplementary Table 1. Ability of MIMESIS-2 to correctly model West Nile virus (WNV) human confirmed cases. Reported are the absolute numbers of municipalities with observed (OBS) and modelled (MOD) cases, for each of six possible ranges of infected humans values (IH)

IH	OBS	MOD
1-3	67	66
4-10	50	52
11-20	20	19
21-30	9	8
31-40	3	2
>40	5	6
SUM	154	153

Supplementary Table 2. List of parameters of the MIMESIS-2 model.

Parameter	Value	Interpretation	Reference
b_L	$b_L(T) = \frac{0.7988}{1 + 1.231 * e^{-0.187(T-20)}}$	Birth rate, larvae	1
m_L	$m_L(T) = 0.0025 * T^2 - 0.094 * T + 1.0257$	Mortality rate, larvae	1
b_M	$b_M(T) = \frac{b_L(T)}{10}$	Birth rate, mosquitoes	1
m_M	$m_M(T) = \frac{m_L(T)}{10}$	Mortality rate, mosquitoes	1
γ_M	$\gamma_M(T) = 0.0093 * T - 0.1352, T > 15°C$ $\gamma_M(T) = 0, T \leq 15°C$	Rate with $1/\gamma_M$ extrinsic-incubation period	1
δ_M	$\delta_M(D) = 1 - \frac{1}{1 + 1775.7 * e^{1.559(D-18.177)}}$	Fraction mosquitoes non-diapausing	1
k	$k(T) = \frac{0.344}{1 + 1.231 * e^{0.184(T-20)}}$	Mosquitoes biting rate	1
b_B	$b_B(d) = \frac{\left(\frac{d}{\beta}\right)^{\alpha-1} * e^{-\frac{d}{\beta}}}{\beta\Gamma(\alpha)}$	Birth rate, birds	1
p_M	$p_M(T) = \begin{cases} 0.02 \; if \; T \leq 19.25 \text{ °C} \\ 0.04 \; if \; 19.25 < T < 21.75 \text{ °C} \\ 0.06 \; if \; 21.75 < T < 24.25 \text{ °C} \\ 0.20 \; if \; 24.25 < T < 26.75 \text{ °C} \\ 0.34 \; if \; T > 26.75 \text{ °C} \end{cases}$	WVV transmission probability (mosquitoes to birds)	2
m_B	0.00034	Mortality rate, birds	3
p_B	$p_B(T) = \begin{cases} 0.28 \; if \; T \leq 19.25 \text{ °C} \\ 0.39 \; if \; 19.25 < T < 21.75 \text{ °C} \\ 0.50 \; if \; 21.75 < T < 24.25 \text{ °C} \\ 0.56 \; if \; 24.25 < T < 26.75 \text{ °C} \\ 0.62 \; if \; T > 26.75 \text{ °C} \end{cases}$	WNV transmission probability (birds to mosquitoes)	2
α_B	0.4	Removal rate, birds	3
γ_B	1.0	Rate with $1/\gamma_M$ intrinsic incubation period	3
ν_B	0.05	Fraction birds dying due to infection	See methods section
ψ_B	0.7	Fraction of bites given from mosquitoes to birds acting as amplifying hosts	4
b_H	0.000033	Birth rate, humans	5
m_H	0.000034	Mortality rate, humans	5
r_H	$r_H = b_H - m_H$	Reproduction rate, humans	5
α_H	0.5	Removal rate, humans	3
γ_H	0.25	Transition rate from exposed to infected, humans	3
ν_H	0.004	Fraction humans dying due to infections	3

ψ_H	0.016	Fraction of bites given from mosquitoes to humans that, after the virus is transmitted, will lead to reported human WNV cases	[4]
A_m	A_m(municipality)	Area of municipality m	[5]
b_d	b_d(municipality)	Birds density	After calibration
$S_{B,0}$	$S_{B,0}$(municipality)= A_m(municipality) * b_d(municipality)	Initial population of susceptible birds	See methods section
$I_{M,0}$	$I_{M,0}$ (municipality, year)	Initial population of infected mosquitoes	After calibration
$N_{M,min}$	$N_{M,min}$(municipality)=c*50,000/3600 * A_m(municipality)	Minimum number of non-diapausing mosquitoes	After calibration
$S_{M,0}$	$S_{M,0}$(municipality)= $N_{M,min}$	Initial population of susceptible mosquitoes	See methods section
K_B	K_B(municipality)=1.2 * $S_{B,0}$	Carrying capacity, birds	See methods section
K_M	K_M(municipality)= K_B(municipality)*30	Carrying capacity, mosquitoes	[6]
$N_{H,0}$	$N_{H,0}$(municipality)	Initial number of susceptible humans	[5]

Supplementary Table 3. List of abbreviation included in the manuscript.

Abbreviation	Description
WNV	West Nile virus
WNF	West Nile fever
WNND	West Nile neuro-invasive disease
MIMESIS	Spatial dynaMIcal Model for wESt nIle virus
HCDCP	Hellenic Centre for Disease Control and Prevention
ECMWF	European Centre for Medium Weather Forecast
L_M	Larvae Mosquitoes
S_M	Susceptible Mosquitoes
E_M	Exposed Mosquitoes
I_M	Infectious Mosquitoes
S_B	Susceptible Birds
E_B	Exposed Birds
I_B	Infectious Birds
R_B	Recovered Birds
D_B	Dead Birds
S_H	Susceptible Humans
E_H	Exposed Humans
I_H	Infectious Humans
R_H	Recovered Humans
D_H	Dead Humans
MSE	Mean Square Error
POD	Probability of Detection
MIS	Miss Rate
FAR	False-alarm rate
CSI	Critical Success Index
IH	Infected Humans (annual aggregation at each municipality)
IH_{MOD}	Modelled IH
IH_{OBS}	Observed IH
WY	Week of the year with first human WNV appearance (at each municipalty)
WY_{MOD}	Modelled WY
WY_{OBS}	Observed WY
SD	Standard Deviation

Supplementary Figures

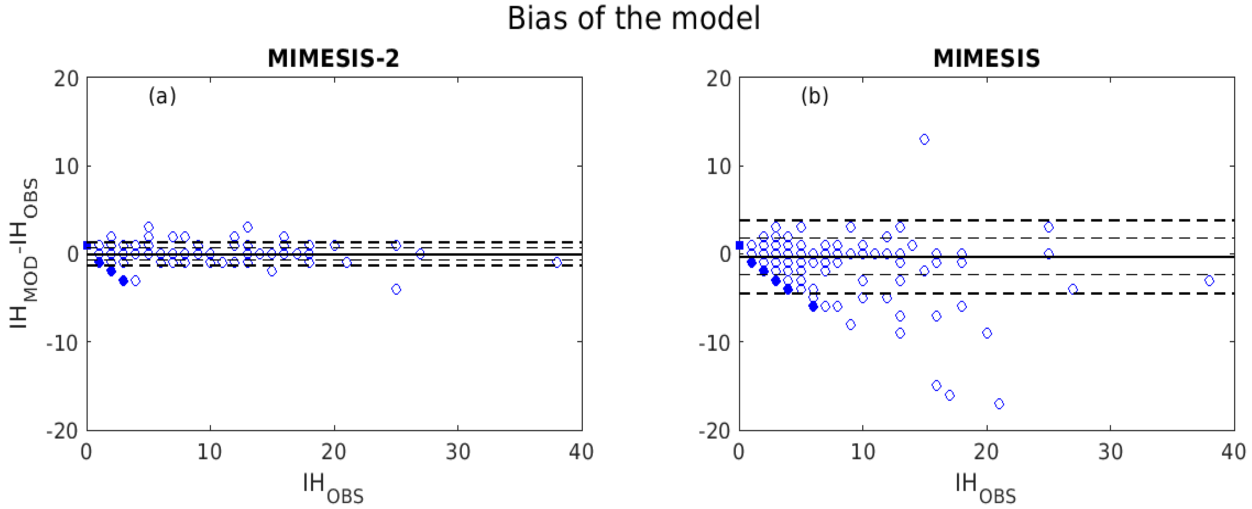

Supplementary Figure 1. (a) For MIMESIS-2, difference between yearly modelled (IH_{MOD}) and observed (IH_{OBS}) human infections, for each municipality and year. True negative cases (3,514) are excluded from the plot. Of the remaining 386 hits are depicted with empty circles, misses with filled circles, and false alarms while filled squares. The filled black line represents the mean bias, while the dashed lines represents the ±standard deviation and ±2 standard deviations. (b) Same quantities for MIMESIS.

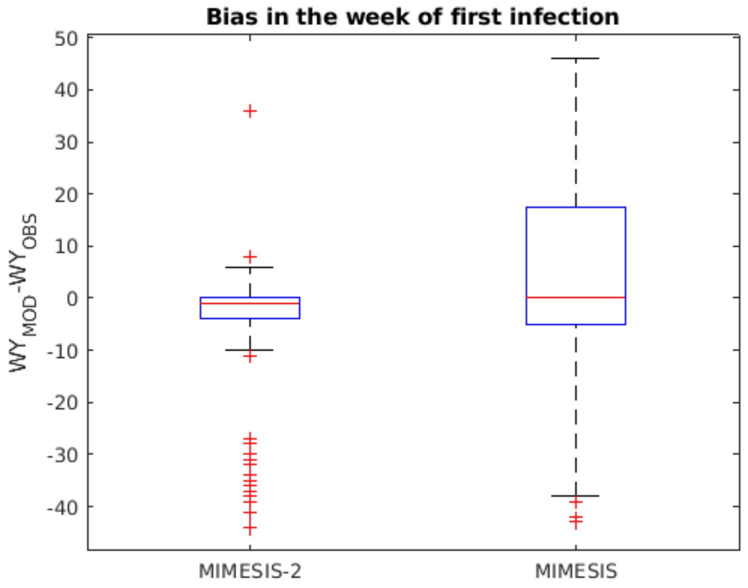

Supplementary Figure 2. Boxplots of the differences between the modelled (WY_{MOD}) and observed (WY_{OBS}) week of first appearance for MIMESIS-2 and MIMESIS across all years and municipalities. True negatives excluded.

References

1 Rubel, F. *et al.* Explaining Usutu virus dynamics in Austria: model development and calibration. *Prev Vet Med* **85**, 166-186, doi:10.1016/j.prevetmed.2008.01.006 (2008).

2 Vogels, C. B., Fros, J. J., Göertz, G. P., Pijlman, G. P. & Koenraadt, C. J. Vector competence of northern European Culex pipiens biotypes and hybrids for West Nile virus is differentially affected by temperature. *Parasit Vectors* **9**, 393, doi:10.1186/s13071-016-1677-0 (2016).

3 Laperriere, V., Brugger, K. & Rubel, F. Simulation of the seasonal cycles of bird, equine and human West Nile virus cases. *Prev Vet Med* **98**, 99-110, doi:10.1016/j.prevetmed.2010.10.013 (2011).

4 Hamer, G. L. *et al.* Host selection by Culex pipiens mosquitoes and West Nile virus amplification. *Am J Trop Med Hyg* **80**, 268-278 (2009).

5 HSA - Hellenic Statistical Authority. <https://www.statistics.gr/en/home/>

6 Angelou, A., Kioutsioukis, I. & Stilianakis, N. I. A climate-dependent spatial epidemiological model for the transmission risk of West Nile virus at local scale. *One Health* **13**, 100330, doi:https://doi.org/10.1016/j.onehlt.2021.100330 (2021).
